# Rabies Postexposure Prophylaxis, Marseille, France, 1994–2005

**DOI:** 10.3201/eid1409.071322

**Published:** 2008-09

**Authors:** Philippe Gautret, Georges Soula, Hamadou Adamou, Marie-José Soavi, Jean Delmont, Yolande Rotivel, Philippe Parola, Philippe Brouqui

**Affiliations:** Hôpital Nord, Assistance Publique– Hôpitaux de Marseille, Marseille, France (P. Gautret, G. Soula, M.-J. Soavi J. Delmont, P. Parola, P. Brouqui); Centre de Formation et Recherche en Médecine et Santé Tropicales, Marseille (G. Soula, J. Delmont); Institut Pasteur, Paris, France (Y. Rotivel); Centre Hospitalier des Armées, Niamey, République du Niger (H. Adamou)

**Keywords:** Rabies, postexposure prophylaxis, France, dispatch

## Abstract

The administration of human rabies postexposure prophylaxis near Marseille (southern France) has changed since the eradication of terrestrial mammal rabies in 2001. Most injuries were associated with indigenous dogs; rabies vaccine was overprescribed. We suggest that the World Health Organization guidelines be adapted for countries free of terrestrial mammal rabies.

The last case of human rabies acquired in France was reported in 1924, and rabies was officially declared eliminated in terrestrial mammals in 2001 (*1*). However, confirmed rabid dogs from North Africa have been imported into France (*2*,*3*), and indigenous bats have been regularly found to be infected by rabies-related viruses (*4*). Marseille is the main international seaport in southern France; it handles heavy daily maritime traffic from North Africa, where numerous human cases are reported in relation with rabid dog bites. Management of patients exposed to these potentially rabid animals poses specific problems, and the decision to prescribe rabies vaccine and/or rabies immunoglobulin depends on the origin of the animal, as it does in the United Kingdom (*5*).

## The Study

From 1994 through 2005, epidemiologic data on animal-related injuries and associated postexposure prophylaxis (PEP) treatment were prospectively collected for Marseille Rabies Treatment Centre patients. Only patients who had been injured in France were selected; rabies PEP for travelers who were injured abroad is detailed elsewhere (*6*). Of the 4,965 eligible patients, 4,367 were outpatients or inpatients (192–488/year), and from 2001 through 2005, a total of 598 were managed by teleconsultation only because their exposure risk was considered to be zero.

**Figure 1:**
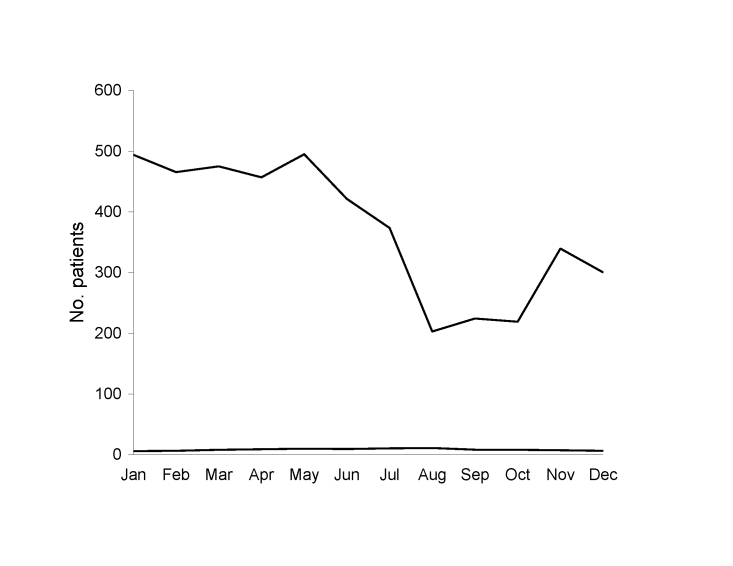
Number of injured patients per year seeking care for rabies postexposure prophylaxis, Marseille Centre, Marseille, France, 1994–2005.

**Figure 2:**
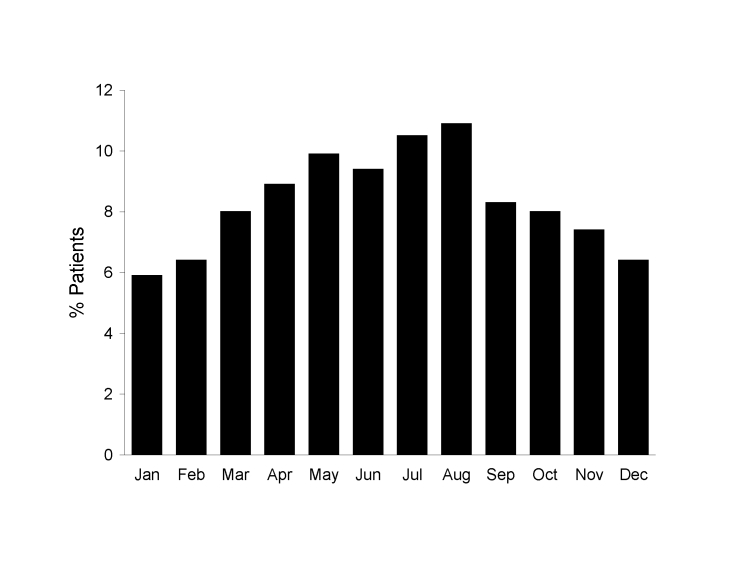
Average proportions of injured patients seeking care for rabies postexposure prophylaxis, by month, Marseille Centre, Marseille, France, 1994–2005.

The number of inpatients and outpatients decreased markedly from 1999 to 2001 **(**Figure 1), which is consistent with the general decrease in the number of PEP treatments in France after the elimination of terrestrial mammal rabies (*7*). Furthermore, prescreening of persons by telephone also contributed to this decrease. The increase observed during 2004–2005 is likely an effect of the international alert in relation to the cases of rabid dogs imported from Morocco; these cases were intensively reported by the French media. The proportion of animal-related injuries tended to increase in late spring/early summer (Figure 2), probably as a result of increased outdoor activities in southern France, which makes contact with animals more likely.

The overall annual incidence of injured patients seeking care for rabies PEP was 16/100,000, which is consistent with incidence recently reported in United States (*8**,**9*) (where rabies is enzootic in bats and raccoons) but far less than that reported in recently available studies from the canine rabies–endemic countries of Turkey (467/100,000) (*10*) and India (1,700/100,000) (*11*). The overall mean annual incidence in our study was 20/100,000 before 2001 and 11/100,000 after 2001.

Dogs accounted for 81.2% of all injuries. By contrast, a recent study on pet demographics in France indicated that dog and cat populations are nearly similar at 8.51 million and 9.94 million, respectively (*12*). This finding suggests that dogs, more often than cats, are responsible for severe injuries that lead persons to seek care for rabies PEP. The mean annual incidence of animal-related injuries was lower in rural than in urban communities (Technical Appendix). Because an estimation of the dog population in France indicated that 41% live in urban areas (*12*), our results suggest that a high human population density increases the probability of human–dog interactions and risk for injuries.

Among patients seeking care for rabies PEP, most were male (male:female ratio 1.49) and mean age was 31.5 (median 29, range 0–96) years. Patients <15 years of age represented 26% of the cohort. The likelihood for animal-related injuries among male patients was also dependant on the animal species involved; dogs, bats, and monkeys accounted for most injuries (Technical Appendix). In contrast, female patients were more likely to be injured by cats, a finding consistent with previous reports (*13*).

The mean time between injury and consultation was 2.6 days (range 0–365 days) and did not statistically vary by sex or age group. Time was longer in patients who were injured by bats (p<10^–6^, online Technical Appendix), probably because most bat bites are nonpainful and considered benign by patients who ignore the risk for rabies after bat contact.

Most injured persons experienced severe contact with animals (95.1%), categorized by the World Health Organization (WHO) as category III (*14*). Most injuries were on the limbs (Technical Appendix).

Animals were available for observation by a veterinarian in 1,441 cases (33%). Rabies testing of animal is not available in southern France, and animals from this region should be sent to the Rabies Laboratory at the Pasteur Institute in Paris, which was done for 89 cases, of which 20 cases were related to a confirmed rabid source from Africa or the Middle East (Table 1).

**Table 1:** Characteristics of postexposure prophylaxis for patients exposed to confirmed rabies source, Marseille, France, 1994–2005

Date of exposure	No. treatments	Confirmed source	Location of exposure, France
1994 Jul	1	Fox	Northeast
1995 Nov	14	Dog*	Southeast
1998 May/Jun	2	Dog†	Southeast
2004 Aug	3	Dog‡	Southwest

*Imported from Burkina Faso. †Unknown origin; rabid strain close to Egyptian isolates. ‡Imported from Morocco (187 treatments were given in France; most in Bordeaux Centre).

The proportion of patients who received treatment increased from 42% during 1994–2000 to 84.3% during 2001–2005 (p<10^–6^) as a result of prescreening by telephone (Table 2). Since 2001, when the animal was not available for surveillance by a veterinarian (which includes numerous cases in which the animal was available for observation by its owner), complete treatment was given to most (89%) patients. Rabies immunoglobulin was provided to 3.2% of these patients, most of whom were injured by bats or severely injured by domestic animals when the owner was not identified or when surveillance of the responsible animal was not possible. No cases of rabies infection were identified in treated persons.

**Table 2:** Treatment for injured patients seeking care for rabies postexposure prophylaxis, by animal rabies status, Marseille, France, 1994–2005*

Patient receipt of PEP*	Animal status						
	1994–2000, no. (%)				2001–2005, no. (%)		
	Unknown†	Rabid‡	Not rabid§		Unknown†	Rabid‡	Not rabid§
Total	1,916 (61.5)	21 (0.6)	1,185 (37.9)		911 (73.2)	5 (0.4)	329 (26.4)
Unknown	0	0	0		4 (0.5)	0	0
None	761 (39.7)	1 (4.8)	1,048 (88.4)		34 (3.7)	0	158 (48.0)
Treatment completed	1,000 (52.2)	20 (95.2)	19 (1.6)		811 (89.0)	5 (100)	45 (13.7)
Treatment stopped	42 (2.2)	0	117 (9.9)		3 (0.3)	0	126 (38.3)
Lost to follow-up	113 (5.9)	0	1 (0.1)		59 (6.5)	0	0
RIG	2 (0.2)	20 (95.2)	1 (0.1)		29 (3.2)	0	14 (4.3)

*PEP, postexposure prophylaxis; RIG, rabies immunoglobulin (% as proportion of treatments including rabies PEP). 1994–2000, n = 3,122; 2001–2005,  n = 1,245. †Animal not available for observation by a veterinarian (including cases where animal was available for observation by its owner). ‡Animal proven to be rabid by laboratory testing or considered rabid upon clinical criteria. §Animal proven to be not rabid by laboratory testing or after 2 weeks of observation by a veterinarian.

## Conclusions

Our rabies PEP data are consistent with data from the national French Referral Center (*7*). The therapeutic approach in France is partly in accordance with WHO general recommendations that in rabies-free areas where adequate rabies surveillance is in effect, rabies PEP may not be required, depending on the outcome of a risk assessment conducted by a medical expert (*14*). Systematic rabies PEP is cost-effective and safe but should not be used if the biting animal is unlikely to be rabid. Furthermore, treating a patient with only vaccine when the animal is under observation could reduce the benefit of further administration of rabies immunoglobulin if the time between vaccination and rabies immunoglobulin injection is >7 days (*15*). If the treatment cannot be delayed, it should include both vaccination and rabies immunoglobulin in cases of category III injury. From 2001 through 2005, not vaccinating the patient when the animal was under observation by its owner or a veterinarian would have represented an overall savings of 177,600 Euros.

To minimize overprescription of vaccination for rabies PEP when treatment may be unjustified, we recommend delaying the initiation of rabies treatment in injuries involving an apparently healthy indigenous dog or cat that can be kept under veterinary or animal-owner observation for 2 weeks, which is the maximum rabies incubation time in these animals. Doing so would result in no rabies treatment for almost all such patients. However, when animals are not available for observation, complete rabies PEP treatment should be initiated. Given the risk for importation of rabid animals from nearby rabies-endemic countries, immediate rabies PEP treatment according to WHO guidelines should be given when the following are involved: indigenous bats; animals illegally imported from rabies-endemic countries; or animals found in railway stations, trains, or other ports of entry. If the animal is suspected of being rabid at the time of exposure, confirmatory testing should be conducted (Technical Appendix). All travelers visiting countries where rabies is enzootic should be informed about the risks of bringing animals back to their home country and about the WHO recommendations regarding rabies vaccination of imported animals (*14*).

## Supplementary Material

Technical Appendix Rabies Postexposure Prophylaxis, Marseille, France, 1994-2005

## References

[1] Rotivel Y, Goudal M, Simons de Fanti A. Human rabies prophylactics: the French experience. Vaccine. 2003;21:710–5. 10.1016/S0264-410X(02)00586-8 12531346

[2] Servas V, Mailles A, Neau D, Castor C, Manetti A, Fouquet E, An imported case of canine rabies in Aquitaine: investigation and management of the contacts at risk, August 2004–Marsh 2005. Eurosurveill. 2005;10:222–5 [cited 2008 Jul 17]. Available from http://www.eurosurveillance.org/ViewArticle.aspx?ArticleId=578 16371687

[3] Canine rabies in France: alert. ProMED. 2008 Mar 7 [cited 2008 Jul 17]. Available from http://www.promedmail.org, archive no. 20080307.0938

[4] Picard-Meyer E, Barrat J, Tissot E, Verdot A, Patron C, Barrat MJ, Bat rabies surveillance in France, from 1989 through May 2005. Dev Biol (Basel). 2006;125:283–8. 16878486

[5] Health Protection Agency. Protocol for the health protection management of rabies. Health protection Scotland. Updated May 2007 [cited 2008 Jul 17]. Available from http://www.documents.hps.scot.nhs.uk/giz/rabies/protocol-management-rabies-2007-05.pdf

[6] Gautret P, Shaw M, Gazin P, Soula G, Delmont J, Parola P, Rabies post-exposure prophylaxis in returned injured travelers from France, Australia and New Zealand: a retrospective study. J Travel Med. 2008;15:25–30. 18217866 10.1111/j.1708-8305.2007.00164.x

[7] Institut Pasteur. Epidémiologie et prophylaxie de la rage humaine en France, 2006. Bull CNRR. 2006;25:1–12 [cited 2008 Jul 17]. Available from http://www.pasteur.fr/sante/clre/cadrecnr/rage/Bull2006.pdf

[8] O’Bell SA, McQuiston J, Fergusson SC, Williams LA. Human rabies exposures and postexposure prophylaxis in South Carolina, 1993–2002. Public Health Rep. 2006;121:197–202. 16528954 10.1177/003335490612100215 PMC1525274

[9] Blanton JD, Bowden N, Eidson M, Wyatt JD, Hanlon CA. Rabies postexposure prophylaxis, New York, 1995–2000. Emerg Infect Dis. 2005;11:1921–7. 16485480 10.3201/eid1112.041278 PMC3367620

[10] Kilic B, Unal B, Semin S, Konackci SK. An important public health problem: rabies suspected bites and post-exposure prophylaxis in a health district in Turkey. Int J Infect Dis. 2006;10:248–54. 10.1016/j.ijid.2005.05.010 16458565

[11] Sudarshan MK, Mahendra BJ, Narayan DH. A community survey of dog bites, anti-rabies treatment, rabies and dog population management in Bangalore city. J Commun Dis. 2001;33:245–51. 12561501

[12] Fabricants d’Aliments Préparés pour Chiens, Chats, Oiseaux. La population animale – enquête FACCO/TNS Sofres. FACCO Magazine. 2005;31:2–9 [cited 2008 Jul 17]. Available from http://www.facco.fr/enseignement.htm

[13] Gautret P, Schwartz E, Shaw M, Soula G, Gazin P, Delmont J, J. Animal-associated injuries and related diseases among returned travelers: a review of the GeoSentinel Surveillance Network. Vaccine. 2007;25:2656–63. 10.1016/j.vaccine.2006.12.034 17234310

[14] World Health Organization. Consultation on rabies: first report, October 2004. Technical report series 931. Geneva: The Organization; 2005. 16485446

[15] Khawplod P, Wilde H, Chomchey P, Benjavongkulchai M, Yenmuang W, Chaiyabutr N, What is an acceptable delay in rabies immune globulin administration when vaccine alone had been given previously? Vaccine. 1996;14:389–91. 10.1016/0264-410X(95)00213-K 8735549

