# Supplementary material for: Rabies Postexposure Prophylaxis, Marseille, France, 1994–2005
Source: Emerg Infect Dis. 2008 Sep;14(9):1452–4. doi: 10.3201/eid1409.071322 (PMC2603096; doi:10.3201/eid1409.071322)
Supplement: Technical Appendix — Rabies Postexposure Prophylaxis, Marseille, France, 1994-2005 [file 07-1322_Techapp-s1.pdf]

Rabies Postexposure Prophylaxis, Marseille, France, 1994–2005

Technical Appendix

Technical Appendix Table 1. Annual incidence of injured patients seeking rabies postexposure prophylaxis treatment according to human population density in the Department of Bouches du Rhône, France, 1994–2005*

Human population/ commune	Population	No. communes	Injured patients seeking rabies PEP treatment			
			1994–2000		2001–2005	
			No. cases	Incidence†	No. cases	Incidence†
Unknown	–	–	21	–	31	–
<5,000	178,306	68	124	10.1 (1.67495)	45	6.0 (2.18432)
5,000–14,999	315,569	37	291	13.2 (1.40213)	130	8.4 (1.37270)
≥15,000	1,344,344	15	2116	17.2 (2.75567)	805	9.2 (1.06092)

*PEP, postexposure prophylaxis.
†Mean annual incidence/100,000 individuals (standard error).

Technical Appendix Table 2. Injured patients seeking rabies postexposure prophylaxis treatment, by animal species, Marseille, France, 1994–2005

Animal	Patients, no. (%)	M:F ratio	Interval, d*
Bat	46 (1.1)	2.07	4.6
Dog	3,547 (81.2)	1.70	2.1
Cat	497 (11.4)	0.71	2.7
Monkey	30 (0.7)	1.73	2.3
Rodent	166 (3.8)	1.00	0.7
Other	81 (1.8)	1.03	3.3
All	4,367 (100)	1.49	2.6

*Interval, mean time between injury and clinic visit.

Technical Appendix Table 3. Type of contact and site of injury in patients seeking care for rabies postexposure prophylaxis, Marseille, France, 1994–2005*

Variable	No. (%)
Type of contact with suspected or confirmed rabid animal	
Unknown	12 (0.3)
Category I	38 (0.9)
Category II	166 (3.8)
Category III	4,151 (95.1)
Body site of injury	
Unknown	2 (0.1)
Upper limbs	2,038 (46.7)
Lower limbs	1,593 (36.5)
Head	369 (8.4)
Trunk	115 (2.6)
Multiple	250 (5.7)

*Category I, touching or feeding animals, licks on intact skin; category II, nibbling of uncovered skin, minor scratches or abrasions without bleeding; category III, single or multiple transdermal bites or scratches, licks on broken skin or mucous membranes, contact with bats.

Technical Appendix Table 4. Recommended postexposure prophylaxis (adaptation of World Health Organization recommendations)

				Recommendation for rabies-free countries‡	
Category	Type of contact*	Type of exposure	Recommendation for rabies-endemic countries†	Indigenous wild animals/apparently healthy and indigenous cat and dog kept under observation for 10 days	Imported animals§ and cats and dogs found in railway station, train or harbor
I	Touching or feeding of animals, licks on intact skin	None	None, if reliable case history is available	None, if reliable case history is available	
II	Nibbling of uncovered skin, minor scratches or abrasions without bleeding	Minor	Administer vaccine immediately¶	None, if reliable case history is available	Administer vaccine immediately¶
III	Single or multiple transdermal bites or scratches, licks on broken skin, contamination of mucous membrane with saliva	Severe	Administer rabies immunoglobulin and vaccine immediately¶	None, if reliable case history is available	Administer rabies immunoglobulin and vaccine immediately#
I, II, III	Exposure to bats	Severe	Administer rabies immunoglobulin and vaccine immediately¶		

*Contact with a suspect or confirmed rabid domestic or wild animal or animal unavailable for testing.
†In all cases: wound cleansing for a minimum of 15 min, using water and soap and virucidal antiseptic (povidone-iodine or ethanol).
‡Countries in which terrestrial mammals are rabies free, but risk exists for importation of rabid animals from nearby rabies-endemic countries.
§Imported cats and dog from rabies endemic areas and new exotic pets, e.g., raccoons, ferrets, squirrels.
¶Stop treatment if animal remains healthy throughout a 10-d observation period (cats and dogs) or if animal is proven to be negative for rabies by a reliable laboratory using appropriate diagnostic techniques.
